# Selective extraction of potassium from raw nepheline materials

**DOI:** 10.1016/j.heliyon.2024.e29461

**Published:** 2024-04-13

**Authors:** Nazym Akhmadiyeva, Sergey Gladyshev, Rinat Abdulvaliyev, Bulat Sukurov, Leila Amanzholova

**Affiliations:** Institute of Metallurgy and Ore Beneficiation, Satbayev University, Almaty, Kazakhstan

**Keywords:** Technology, Nepheline, Potassium, Leaching, Alumina

## Abstract

The global aluminium industry is ever-changing and primarily relies on bauxite as its traditional source. However, due to the finite reserves of processed bauxite, alternative sources need to be found. Nephelines have emerged as potential alumina sources. In addition to alumina, nepheline processing can yield marketable products such as soda, potash, cement, and rare metals.

The objective of this study was to identify the ideal conditions for selectively extracting potassium from alkaline leaching solutions while separating it from sodium alkali, aluminium, and silicon during the comprehensive processing of nepheline syenites from the Kubasadyr deposit. This approach involved a two-stage hydrothermal leaching process with an incremental calcium addition. Under optimal conditions, the potassium extraction in the alkaline solution reached 93.91 %. The resulting leaching solution served as a feedstock for potassium sulfate production through the crystallization method. The preliminary selective separation of potassium played a crucial role in mitigating its adverse effects on the extraction of aluminium, which is the primary processed product; thus, the economic viability of production was enhanced.

## 1 Introduction

In the aluminium industry, the primary raw material for alumina production is bauxite, which is processed through the Bayer process. Due to the limited reserves of Bayer bauxite, nepheline, which is a nonbauxite raw material high in silica, should be considered as a potential alumina source.

Nepheline-containing raw materials consist of alkaline aluminosilicates, with nepheline, K-feldspars, and coloured minerals being the main components [1]. The products of nepheline processing include alumina, sodium, potassium salts (soda, potash, and potassium sulfate), and cement. Alumina has the most economic significance, and the production of potassium salts is driven by the increasing importance of potassium in fertilizers and agriculture.

Potassium can be obtained from diverse sources, such as seawater, salt deposits, and low-grade minerals. Soluble mineral resources containing potassium are found in numerous regions globally [2,3]. To alleviate the scarcity of potassium salts and diminish the reliance on limited soluble minerals, alternative resources, with a focus on low-grade potassium-bearing ores, are the focus of ongoing research endeavours. Silicate minerals containing potash include potassium feldspar, gluconitic sandstones, nepheline syenites, and mica.

The challenge in extracting potassium from potassium-bearing aluminosilicate ores stems from their intricate structure; in ores, potassium is tightly bound to aluminium and silicon, causing poor water solubility of the potassium cations [[3], [4], [5]]. Standard techniques for breaking down potassium-bearing minerals include acid leaching, thermal decomposition, and hydrothermal alkaline processes [[6], [7], [8]].

The benefit of acid leaching lies in its low leaching temperature (below 120 °C). However, achieving substantial potassium dissolution requires an elevated acid concentration, a high liquid-to-solid ratio, and an extended reaction time. Certain minerals, including muscovite and nepheline syenite, exhibit stability and resistance to dissolution [1,9].

To enhance potassium extraction, sulfuric acid has been employed, and various techniques, such as ultrasonic acid leaching [10], sulfate roasting [[9], [11]], and mechanical activation treatment [4], have been implemented.

The acid leaching method has been shown to be ineffective in achieving complete potassium dissolution from potassium-sodium aluminosilicates, particularly nepheline syenites, owing to their complex structure. Instead, the preferred approach for processing nepheline syenites involves thermal decomposition [12].

In the pursuit of potassium extraction, roasting has been employed, incorporating various additives such as calcium chloride [4,13], resulting in a potassium extraction of 91 % at 900 °C, and a CaCl_2_–NaCl mixture, achieving a potassium extraction of 95.35 % at 800 °C [14]. However, a drawback of thermal decomposition lies in its high energy intensity.

The hydrothermal dissolution of potassium-bearing silicates under alkaline conditions using Ca(OH)_2_–2H_2_O, NaOH, and KOH has been documented [[14], [15], [16], [17], [18], [19], [20], [21], [22], [23]]. Hydrothermal processes provide advantages such as low energy consumption and minimal waste generation. However, notable limitations include the expenses associated with alkaline reagents, autoclave reactors, and extended processing times.

**Fig. 1:**
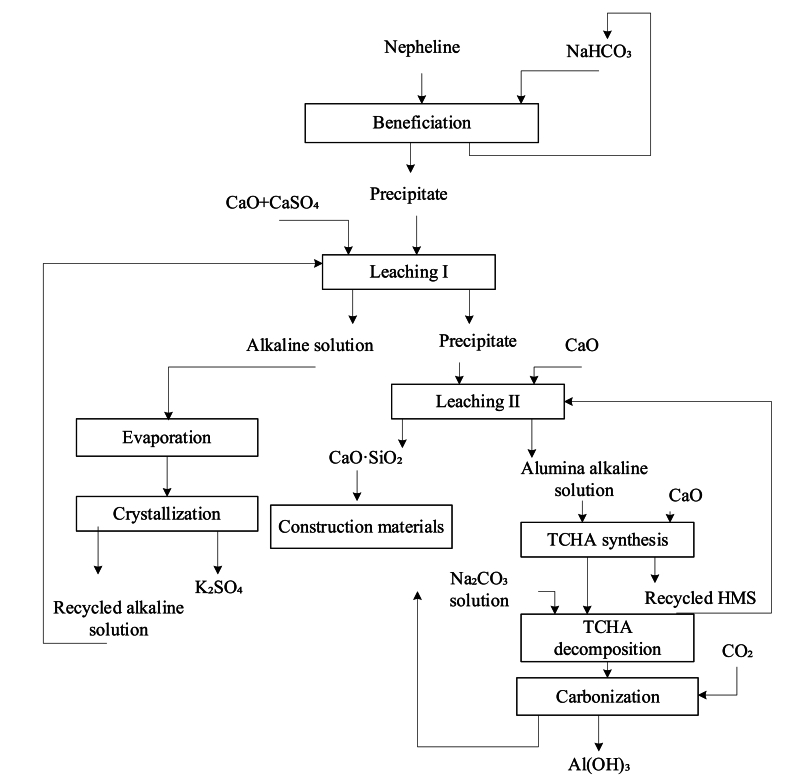
Complex technological scheme for processing nepheline syenites using a fractional calcium additive.

One study [10] detailed the production of potassium salts from potassium-sodium rocks through a comprehensive method involving hydrothermal alkaline treatment followed by the roasting of the insoluble portion (see Fig. 1). Potassium extraction was accomplished through the carbonization of an aluminate leaching solution, yielding 43.5–50.9 % SiO_2_ and 82.1–83.8 % K_2_O. Before carbonization, the aluminate solution underwent desiliconization. Following carbonization and aluminium separation, the solution underwent evaporation, and potassium bicarbonate was crystallized and subsequently converted to potassium carbonate. In the freshly prepared state, the mass ratio of sodium carbonate to potassium carbonate was 1.6–2.0. This technology involved two multistage branches for processing ore leaching intermediate products and included an energy-intensive roasting operation. However, no selective potash was obtained as a result of this processing.

The objective of this research was to identify the optimal conditions for the selective extraction of potassium in an alkaline leaching solution and the separation of potassium from sodium alkali, aluminium, and silicon in the comprehensive processing of nepheline syenites through a hydrothermal method with a fractional introduction of a calcium additive (see Fig. 1).

The proposed technological scheme for the complex technology to process nepheline syenites using a fractional calcium additive includes the following steps.

- Stage I: leaching of nepheline ore in an alkaline solution with the addition of CaO mixed with CaSO_4_ for selective extraction of potassium and part of SiO_2_ into the solution
- Evaporation of the alkaline solution from leaching stage I to obtain K_2_SO_4_ by crystallization
- Stage II: leaching in a high modulus solution (HMS) with a caustic modulus α_k_ of 30.0 determined from the Na_2_O to Al_2_O_3_ ratio, with the addition of calcium oxide necessary for calcium silicate formation to obtain an aluminous alkaline solution with α_k_ = 10.0
- Conversion of the aluminous alkaline solution by synthesis of tricalcium hydroaluminate (TCHA) to bind the aluminium extracted at stage I of leaching and obtain recycled HMS with α_k_ = 30.0 by the following reaction:

(1) Na_2_O·AI_2_O_3_+3 Са(ОН)_2_ + 4H_2_O = 3CaO·AI_2_O_3_·6H_2_O + 2NaOH

- Decomposition of TCHA with soda solution to obtain an aluminate solution for carbonization by the following reaction:

(2) 3CaO·Al_2_O_3_·6H_2_O + Na_2_CO_3_ = 3CaCO_3_ + 2 NaAl(OH)_4_

- Carbonization of the aluminate solution with CO_2-_containing gas to obtain Al(OH)_3_ and recycled Na_2_CO_3_ solution by the following reaction:

(3) 2 NaAl(OH)_4_ + СО_2_ = 2Al(OH)_3_ + Na_2_CO_3_

- Evaporation of a part of the carbonized soda solution to obtain Na_2_CO_3_ by crystallization.

The preliminary selective separation of potassium using the complex technology for processing nepheline syenites is crucial. This process serves to eliminate the adverse impact of potassium alkali on the extraction of aluminium [24], which is the primary product of processing. This step contributes to the economic viability of production by ensuring the efficient extraction of aluminium without being hindered by the presence of the potassium alkali.

## 2 Materials and methods

X-ray fluorescence analysis of the chemical composition was performed on a Venus 200 wave dispersion spectrometer (Panalytical B. V.). Chemical analysis was performed using an Optima 2000 DV inductively coupled plasma optical emission spectrometer (Optima, PerkinElmer). Semiquantitative X-ray phase analysis was performed on a D8 Advance diffractometer (BRUKER) using copper Cu-Kα radiation at an accelerating voltage of 36 kV and a current of 25 mA. Electron probe microanalysis (in energy dispersive spectroscopy mode) with scanning electron microscopy (in backscattered electron image mode) was performed on a JEOL JXA-8230 instrument. Differential thermal analysis (DTA) was performed using an STA 449 F^3^ Jupiter synchronous thermal analysis instrument. Infrared spectroscopy (IRS) spectra were collected using an FT/IR-6X FT-IR spectrometer in the spectral range of 4000–400 cm^−1^. The coarseness was determined with a Winner 2000 laser particle size analyser from the Photocor series, the measurement principle of which involves static and dynamic light scattering.

The spectra were analysed using infrared spectral databases from the KnowItAll software.

**Fig. 2:**
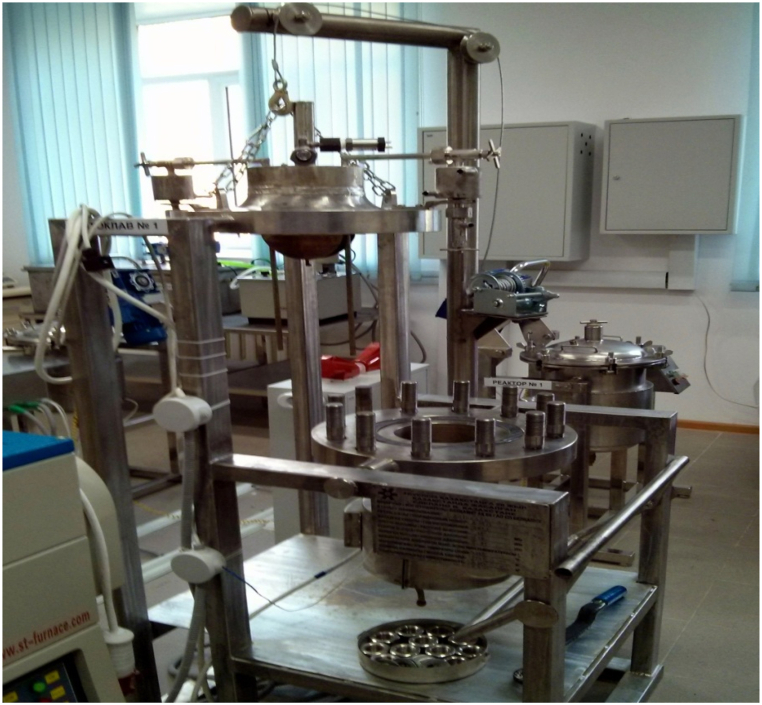
Autoclave picture.

Ore leaching in alkaline solution at temperatures of 200–3300°С was carried out in an autoclave with an overpressure of up to 100 bar, which allowed the temperature to increase to 300°С with the help of an electric heater with a power of 5 kW. The accuracy of temperature maintenance in the autoclave was ±2 °C. The design of the autoclave enabled sampling with quenching throughout the experiment. The design of the autoclave also allows the introduction of the solid phase at a given temperature and stirring by a falling stirrer with a frequency of 0.1–1 Hz. An external view of the autoclave unit is shown in Fig. 2.

## 3 Results and discussion

Nepheline syenites from the Kubasadyr deposit, Republic of Kazakhstan, were used for this research. The chemical composition in wt% was as follows: Al_2_O_3_ – 21.2; SiO_2_ – 55.86; Fe_2_O_3_ – 3.18; CaO – 3.64; Na_2_O – 5.09; K_2_O – 5.52; others – 5.14.

**Fig. 3:**
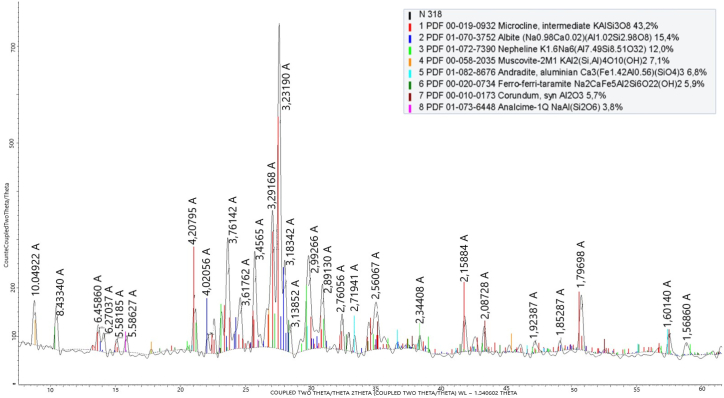
XRF phase composition of the nepheline syenites from the Kubasadyr deposit.

**Fig. 4:**
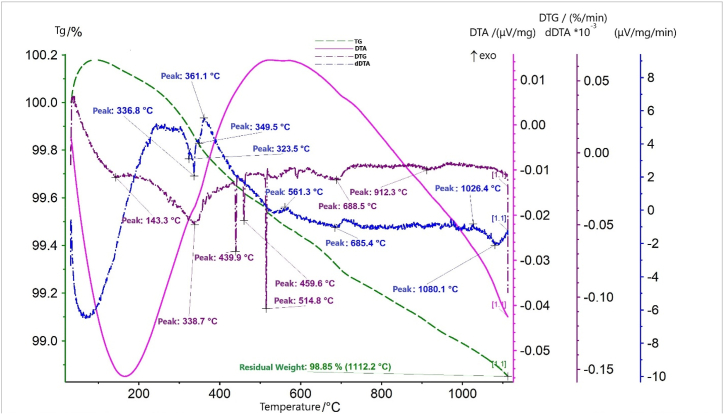
Thermograms of the nepheline syenites.

The phase composition was investigated by X-ray phase analysis (XRF) (Fig. 3) and differential thermal analysis (DTA) (Fig. 4).

According to the results from thermal analysis (Fig. 4), the DTA curve showed numerous endothermic effects of different intensities, with maximum development at 143.3°С, 323.5°С, 336.8°С, 349.5°С, 514.80°С, 685.4°С, and 1080.1°С. Exothermic effects were also observed, with peaks located at 361.1°С, 561.3°С, and 1026.7°С.

Endothermic effects in the temperature range of 100–355 °C were accompanied by a decrease in the weight of the suspension. On the TGA curve, these effects corresponded to the minimum at 338.7 °C, and they were located in the region of their development. Here, the processes of dehydration of iron and aluminium hydroxides occurred. In the temperature region of 355–550 °C on the dDTA curve, no effects were observed; however, on the TGA curve, minima developed in a very narrow temperature range. These minima occurred at 439.9°С, 459.6°С, and 514.8°С. They reflected the dehydration of impurity phases. Most likely, aluminium hydroxides (synthetic bemite, aluminogel) or goethite were present. Additionally, in superposition at 439.9 °C, dehydration of natrolite Na_2_[Al_2_Si_3_O_10_]·2H_2_O and/or analcime Na_2_[Al_2_Si_2_O_6_]·2H_2_O potentially occurred. These phases likely occurred because some of the compounds detected by XRD were thermally inert. Thus, albite, nepheline, corundum, and andradite in the temperature range of 20–1110 °C were not present. Therefore, identification of the phases present in the sample in low quantities was possible.

The exothermic effect with a peak at 361.1 °C was a manifestation of microcline. The combination of this exothermic effect with the exothermic effect with a peak at 1026.7 °C could be interpreted as a manifestation of amorphized SiO_2_. The first peak reflected the transition to the crystalline state, while the second peak reflected the formation of β-cristobalite.

Notably, microcline and orthoclase have the same formula and, during XRD, have the same effects. However, orthoclase is thermally inert. If we assume that in the sample is not microcline but rather orthoclase, the probability that peak 361.1 °C is a manifestation of amorphized SiO_2_ becomes much greater.

The endothermic effect, with a maximum at 685.4 °C, was accompanied by a decrease in the weight of the suspension. The TGA curve showed a minimum at 688.5 °C. Most likely, the process of dehydration of zeolite wairakite-Ca[AlSi_2_O_6_]·2H_2_O was reflected here. The low-intensity, smooth exothermic effect with a peak at 561.3 °C could reflect the crystallization of an impurity of the watered silica gel, SiO_2_-nH_2_O. Considering the endothermic effect, with a maximum at 1080.1 °C on the dDTA curve and a minimum at 912.3 °C on the TGA curve, the mica mineral sericite-KaAl_2_[AlSi_3_O_10_](OH,F)_2_ could be assumed to be present.

Based on the analysis of phase composition, the main feldspar minerals were microcline, albite, muscovite, and nepheline.

**Fig. 5:**
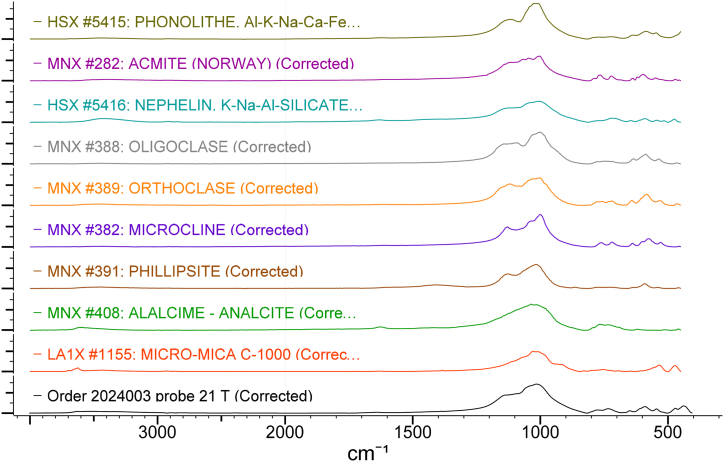
IRS spectrum analysis of nepheline syenite.

According to the results from the IRS analysis (Fig. 5) of the nepheline syenites, the following phases were detected: phonolithe, Al–K–Na–Ca–Fe-silicate, acmite NaFe_3·_Si_2_O_6_, nepheline K–Na–Al-silicate, oligoclase mNaAlSi_3_O_8_ with nCaAl_2_Si_2_O_8_, orthoclase KAlSi_3_O_8_, microcline K (AlSi_3_O_8_), phillipsite (K_2_,Na_2_,Ca) Al_2_Si_4_O_12.4_ 1/2 H_2_O, alalcime – analcite Na (AlSi_2_O_6_)·H_2_O, micro-mica C-1000 (aluminium oxide) 33.15 %; and silica (silicone oxide) 48.40 %.

**Fig. 6:**
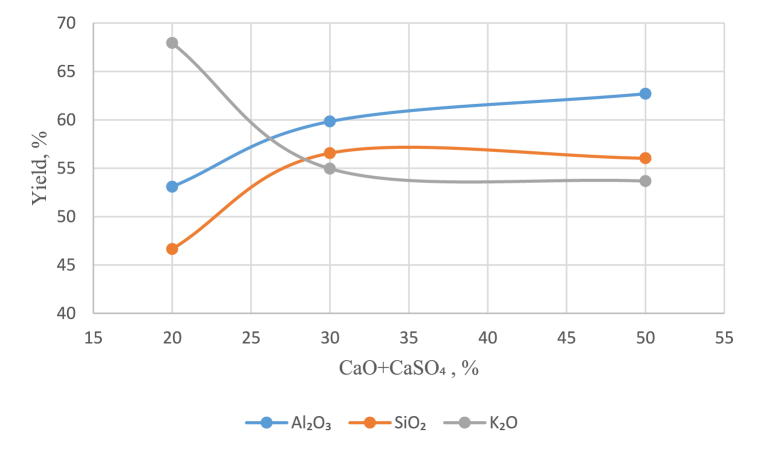
Effect of the amount of added CaO + CaSO₄ on the extraction of compounds in a solution containing 160 g/dm^3^ Na_2_O.

**Fig. 7:**
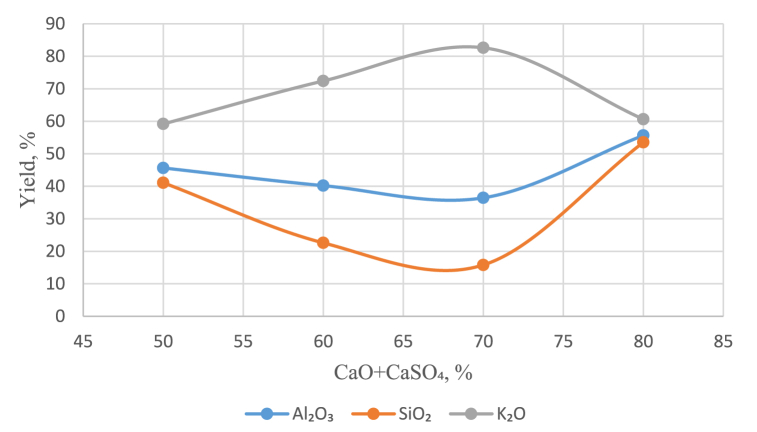
Effect of the amount of added CaO + CaSO₄ on the extraction of compounds in a solution containing 240 g/dm^3^ Na_2_O.

Leaching for the recovery of potassium in solution was carried out in recycled alkaline solutions of Na_2_O (160 and 240 g/dm^3)^ at temperatures of 200 and 280 °C for 30–120 min, and calcium oxide was added as a mixture with CaSO_4_ at a rate of addition of calcium oxide ranging from 20 to 50 % to obtain a pulp molar ratio of CaO:SiO_2_ = 1 (see Table 1, Fig. 6, Fig. 7).

**Table 1:** Extraction of compounds into solution during leaching depending on the amount of calcium added.

СаО+CaSO_4_, %	Leaching conditions					
	160 g/dm^3^, 200°С, 30 min			240 g/dm^3^, 280°С, 90 min		
	Extraction into solution, %					
	Al₂O₃	SiO₂	K₂O	Al₂O₃	SiO₂	K₂O
20	53.09	46.65	67.94	–	–	–
30	59.83	56.56	54.96	–	–	–
50	62.69	56.03	53.67	45.64	41.09	59.17
60	–	–	–	40.2	22.59	72.4
70	–	–	–	36.44	15.79	82.62
80	–	–	–	55.65	53.52	60.64

The addition of calcium sulfate in the leaching of nepheline syenite in the first stage of leaching was performed to obtain potassium sulfate in the Na–K solution, which was further separated by the crystallization using the difference in solubility [24]. The necessary amount of CaSO_4_ added was determined from the calculation of the complete K_2_O extraction into the solution.

The results showed that at a Na_2_O concentration of 160 g/dm^3,^ the degree of potassium extraction into solution was not more than 66 %; at the same time, a high degree of Al₂O₃ and SiO₂ extraction was obtained, which was undesirable.

At a Na_2_O concentration of 240 g/dm^3^, the optimal results were obtained by adding 70 % CaO, which resulted in 82.62 % K_2_O extraction into solution, 15.79 % SiO_2_ extraction and 36.44 % Al_2_O_3_ extraction.

Table 2 shows the results from the X-ray phase analysis of the material composition of the leach sludge, and Table 3 shows the chemical composition of the leach sludge.

**Table 2:** Phase compositions of the sludge after leaching.

Name	Formula	Initial	Leaching conditions						
			160 g/dm^3^, 200°С, 30 min			160 g/dm^3^, 200°С, 30 min			
			Amount of CaO from the stoichiometry CaO:SiO_2_ = 1, %						
			20	30	50	50	60	70	80
Nepheline	K_1·6_Na_6_(Al_7·49_Si_8·51_O_32_)	12.0							
Corundum	Al_2_O_3_	5.7				2.9		2.6	2.6
analcime	NaAl(Si_2_O_6_)	3,8							
Microcline	KAlSi_3_O_8_	43.2	31.6	28.1	20.6	20.0	13.6		20.7
Cancrinite	Na_6·6_Ca_0·9_(Si_6·5_Al_5·5_O_24_) (CO_3_)_1·44_(H_2_O)_2_		23.8	19.3	17.5	11.4	13.4	16.8	
Albite	(Na_0·98_Ca_0.02_)∙ (Al_1·02_Si2·_98_O_8_)	15.4	17.0	19.1	7.0	2.4	2.2	4.0	
Portlandite	Ca(OH_)2_		10.0	14.9	17.4	33.6	32.1	23.9	42.3
Calcium aluminosilicate	CaAl_2_Si_2_O_8_		8.6	6.2	5.0	6.3			
Muscovite	KAl_2_(Si,Al)_4_O_10_(OH)_2_	7.1	4.9	4.5	4.1				
Ferro-ferri-taramite	Na_2_CaFe_5_Al_2_Si_6_O_22_(OH)_2_		4.1	5.4	5.8	1.6	1.1	3.0	1.7
Andradite	Ca_3·027_(Al_0·14_Fe_0.86_)∙ (Al_0·69_Fe_0.31_) ∙ (SiO_4_)_3_	6.8			14.4				
Tobermorite	Ca_2·25_(Si_3_O_7·5_(OH)_1.5_)(H_2_O)				11.1	6.3	22.6	28.3	
Calcite	Ca(CO_3_)				4.2				
Sodium-calcium aluminosilicate sulfide	(N_a6·59_Ca_1.65_)(Al_6_Si_6_O_24_)S_1.58_					6.7	6.9		6.2
Magnetite	Fe_3_O_4_					4.2	4.4		
Mayenite	Ca_12_Al_14_O_33_(H_2_O)						3.4	6.2	
Sodium-calcium silicohydrate	Na_2_Ca_2_Si_2_O_7_·H_2_O							17.1	
Beidelite	(Na,Ca)_0·3_Al_2_(Si,Al)_4_O_10_(OH)_2_ ·xH_2_O								
Wollastonite	CaSiO_3_								8.0
Ternardite	Na_2_SO_4_								5.4
Catoite	(CaO)_3_(Al_2_O_3_)_1·75_(H_2_O)_3.75_								4.6

**Table 3:** The chemical composition of the leach sludge.

Name	Leaching conditions							
	160 g/dm^3^, 200°С, 30 min				160 g/dm^3^, 200°С, 30 min			
	Amount of CaO from the stoichiometry CaO:SiO_2_ = 1, %							
	20	30	50	50		60	70	80
Al₂O₃	9.76	9.47	9.81	7.0		6.67	7.06	4.2
SiO₂	25.39	25.57	32.62	20.65		23.5	25.46	11.98
K₂O	2.22	2.04	0.65	1.58		0.92	0.58	1.12
Na₂O	4.774	5.306	6.898	8.03		11.24	11.34	8.46
Fe₂O₃	2.873	2.841	2.538	1.87		1.54	1.55	1.34
CaO	13.454	16.478	24.398	22.865		17.46	22.015	24.585

According to the results from the X-ray phase analysis, during leaching in a solution containing 160 g/dm^3^ Na_2_O and 200 °C the nepheline phase disappeared, and with increasing amounts of calcium, the content of potassium-containing phases of microcline and muscovite decreased: thus, potassium was released into the solution.

During leaching in a solution containing 240 g/dm^3^ Na_2_O at 280 °C, the phases of nepheline and muscovite also disappeared, and as the amount of calcium added increased to 70 % of the stoichiometric ratio, the amount of microcline decreased, and the amount of potassium extracted from the solution increased. A further increase in the amount of calcium additive had a negative effect on the potassium recovery, with an increase in unreacted CaO, as evidenced by an increase in the proportion of the portlandite phase.

**Fig. 8:**
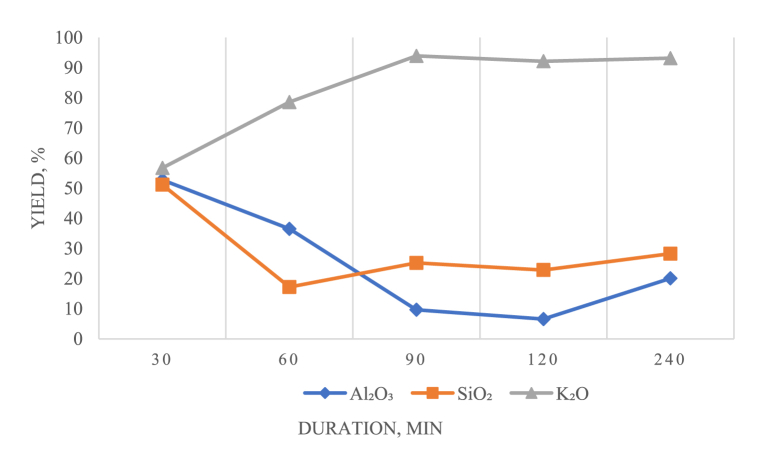
Influence of the leaching duration on the recovery of elements.

The influence of leaching duration was examined in the range of 30–240 min at 280 °C, and the calcium additive was equivalent to 70 % of the stoichiometrically needed value (see Table 4, Fig. 8).

**Table 4:** Influence of the leaching duration on the degree of element extraction.

Duration, minutes	Yield, %			Residue composition, %		
	Al₂O₃	SiO₂	K₂O	Al₂O₃	SiO₂	K₂O
30	52.75	51.15	56.72	6.94	19.53	1.91
60	36.52	17.2	78.58	8.78	31.2	0.89
90	9.66	25.21	93.91	12.35	27.83	0.25
120	6.57	22.84	92.13	11.86	26.66	0.3
240	20.08	28.26	93.15	10.72	26.19	0.28

Analysis of the results showed that with increasing duration of the process, the amount of potassium extracted into the solution increased, and after 90 min, the amount of potassium extracted into the solution remained at 92–93; in contrast, the amount of aluminium extracted decreased. The minimum extraction of Al₂O₃ into solution (6.57 %) was obtained after 120 min of leaching.

Since the aim of the research was to identify the optimal technological conditions for selectively separating potassium alkali from sodium alkali, aluminium, and silicon in the initial stage of fractional calcium additive introduction, the optimal leaching duration was 90 min.

**Fig. 9:**
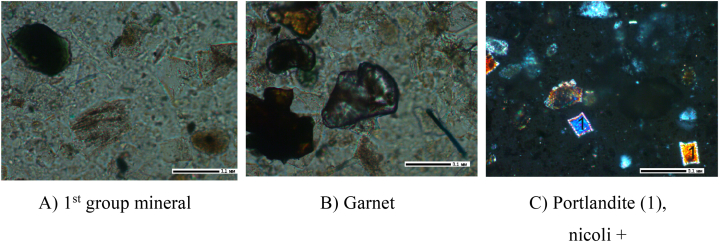
Leach cake after leaching for 30 min, 200 × .

**Fig. 10:**
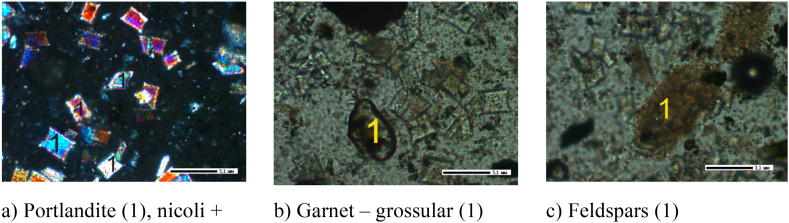
Leach cake after leaching for 60 min, 200 × .

**Fig. 11:**
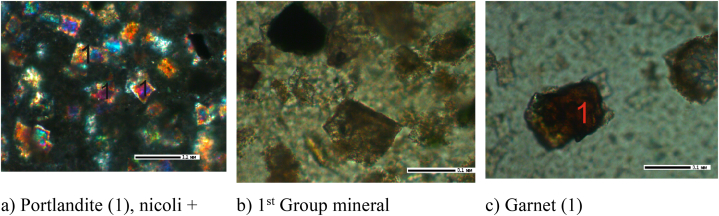
Leach cake after leaching for 90 min, 200 × .

Based on the results obtained from the crystal-optical, X-ray phase, X-ray spectral, and IRS analyses, the primary minerals identified in the cake samples obtained at leaching durations of 30, 60, and 90 min included unreacted nepheline and potassium feldspar. Additionally, albite, portlandite, and garnet were other observed minerals that formed during the leaching process (see Fig. 9, Fig. 10, Fig. 11).

As shown in Fig. 9a, minerals without the characteristic twin structure exhibit a low refractive index below 1.538 (Fig. 9a). These minerals cannot be accurately identified. However, potassium feldspar, albite and nepheline in Group 1 can be classified since they exhibit similar refractive indices. Garnet (Fig. 9b) and portlandite (Fig. 9c) were also found in the sample. Garnet exhibits a high refractive index and is isotropic. Portlandite Ca(OH)^2-^ occurs as a basal plate with a refractive index of 1.574 and is a colourless, uniaxial, negative mineral.

In the cake with a leaching duration of 60 min, portlandite is dominant (Fig. 9a), as well as garnet-grossular (Fig. 9b) and feldspars (Fig. 9c).

Portlandite Ca(OH)_2_ (Fig. 10a) are observed as basal plates with a refractive index of 1.574. The mineral is described as grossular and is a colourless, uniaxial, negative crystal with a high refractive index. Grossular garnet (Fig. 10b) is isotropic; thus, it exhibits the same optical properties in all directions. In this case, the grossular garnet is considered uncoloured or colourless. Feldspars (Group 1 minerals) (Fig. 10c) have a refractive index of less than 1.538.

Portlandites (Fig. 11a), Group I minerals (Fig. 11b) and garnet (Fig. 11c) are observed in the cake with a leaching time of 90 min.

Portlandite Ca(OH)_2_ is observed as a basal plate with a refractive index of 1.574 and is a colourless, uniaxial, negative mineral. Garnet is brown-coloured and isotropic, with a high refractive index.

**Fig. 12:**
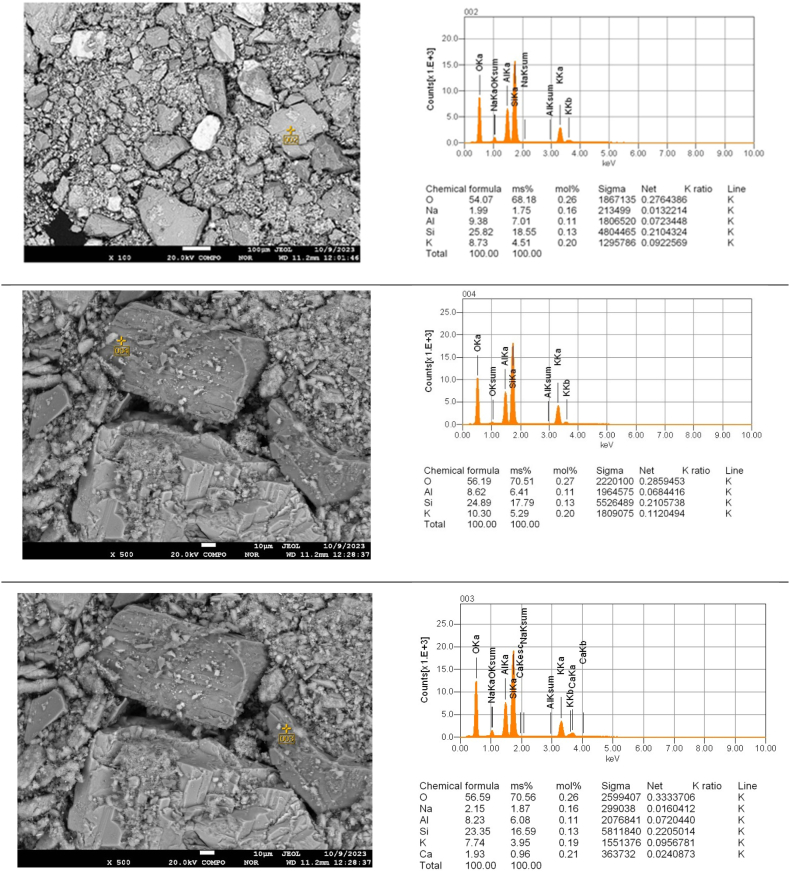
Point EPMA of the particles in the different sections of cake after leaching for 30 min..

**Fig. 13:**
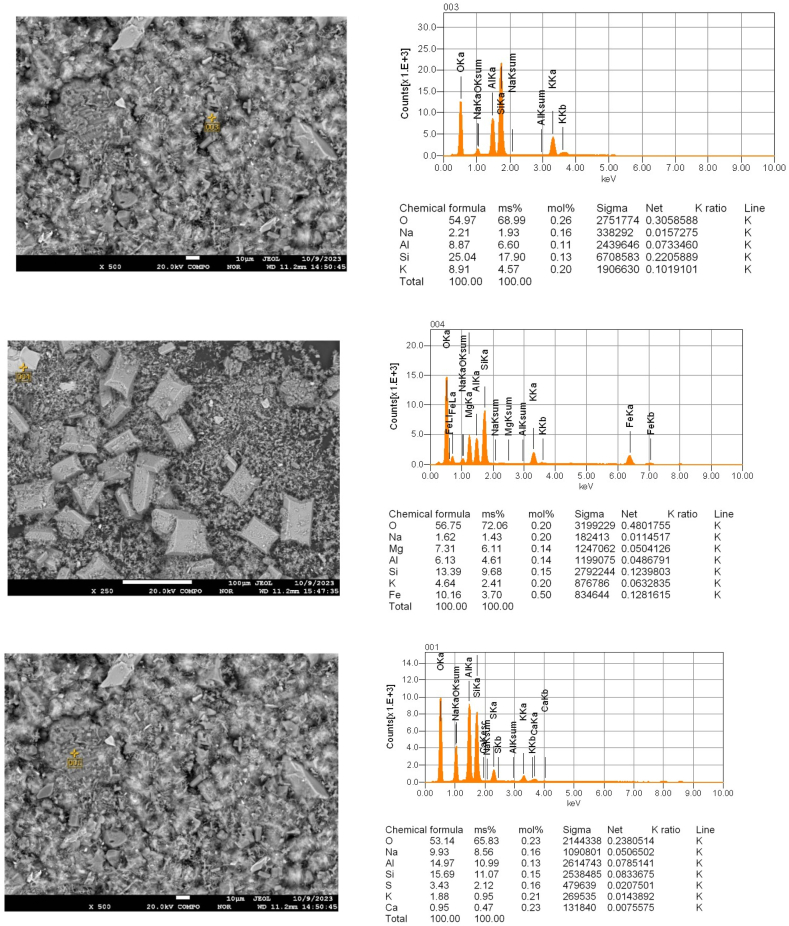
Point EPMA of the particles in the different sections of cake after leaching for 60 min..

**Fig. 14:**
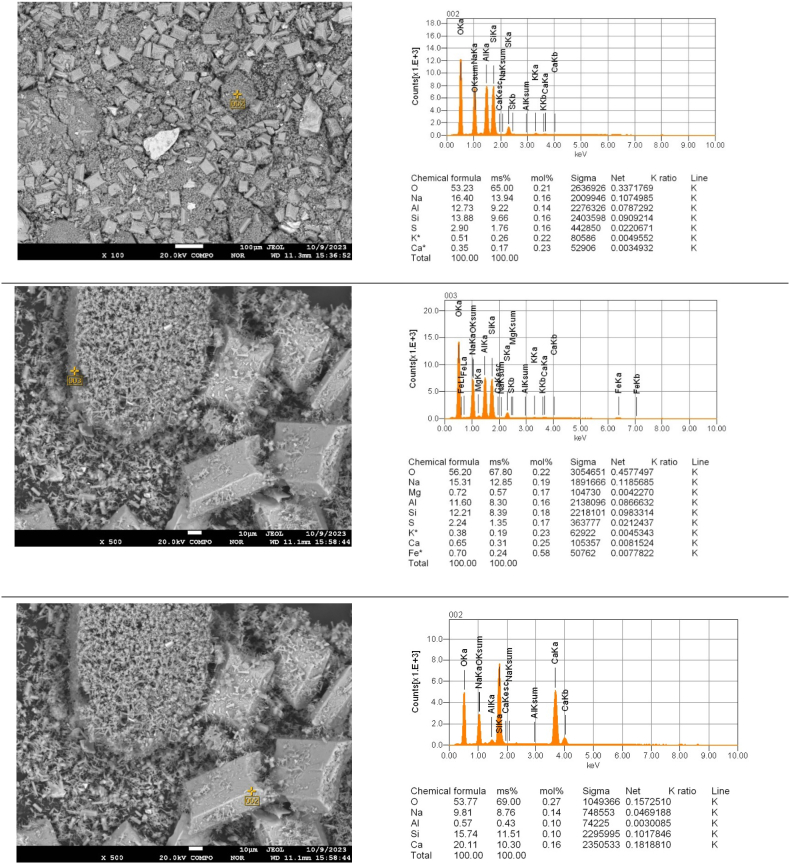
Point EPMA of the particles in the different sections of cake after leaching for 90 min..

Electron probe microanalysis (EPMA) with scanning electron microscopy was used to clarify the elemental composition of the particles in different areas of the cake after leaching as a function of duration (Fig. 12, Fig. 13, Fig. 14).

The EPMA results (Fig. 12, Fig. 13, Fig. 14) indicated that as the duration increased, the quantity of potassium decreased in the various sections.

The dependence of the phase composition of the leach cake with respect to the duration was determined by the XRD method, and the results are provided in Table 5.

**Table 5:** X-ray phase composition of leach cakes depending with respect to duration.

Title	Formula	Duration, min			
		30	60	90	120
Portlandite	Ca(OH)_2_	26.1	12.4		
Microcline	K_0·94_Na_0·06_Al_0·95_Si_3·05_O_8_	25.9			
Beidelite	(Na,Ca)_0·3_Al_2_(Si,Al)_4_O_10_(OH)_2_·xH_2_O	13.4	5.5	4.1	4.7
Albite	Na_0·986_Al_1·005_Si_2·995_O_8_	9.2			
Calcium alumoxide	Ca_4_(Al_6_O_12_)O	7.4			
Pyroxpheroite	Ca_0·94_Fe_6·06_O_21_Si_7_	7.2			
Nepheline	NaAlSiO_4_	6.8			
Ferro-pargasite	NaCa_2_Fe_4_AlSi_6_Al_2_O_22_(OH)_2_	4.1			
Tobermorite	Ca_2·25_(Si_3_O_7·5_(OH)_1.5_)(H_2_O)		48.4		
Hydroxycancrinite	Na_8_(Al_6_Si_6_O_24_)(OH)_2·04_(H_2_O)_2.66_		15.4		
Tamarugite	NaAl(SO_4_)_2_·6H_2_O		5.9		
Bernalite	Fe(OH)_3_(H_2_O)0.25		5.2		
Calcium sulfate	CaSO_4_		5.0		
Corundum	Al_2_O_3_		2.2		
Cancrinite	(Na_6_(Al_6_Si_6_O_24_))(NaOH)_2_(H_2_O)_6_			34.8	43.7
Sodium calcium hydrosilicate	NaCaHSiO_4_			31.3	43.1
Mayenite	(Ca_12_Al_14_O_32_)O_1.32_			6.7	
Pyrite	FeS_2_			5.5	
Ferro-ferri-taramite	Na_2_CaFe_5_Al_2_Si_6_O_22_(OH)_2_			5.2	6.6
Aluminium hydroxide	Al(OH)_3_			4.5	
Mullite	Al_5·65_Si_0·35_O_9.175_			3.9	
Hydrogen-aluminium silicate	H(AlSi_2_)O_6_			2.7	
Moganite	SiO_2_			1.2	
Quartz	SiO_2_				1.9

**Fig. 15:**
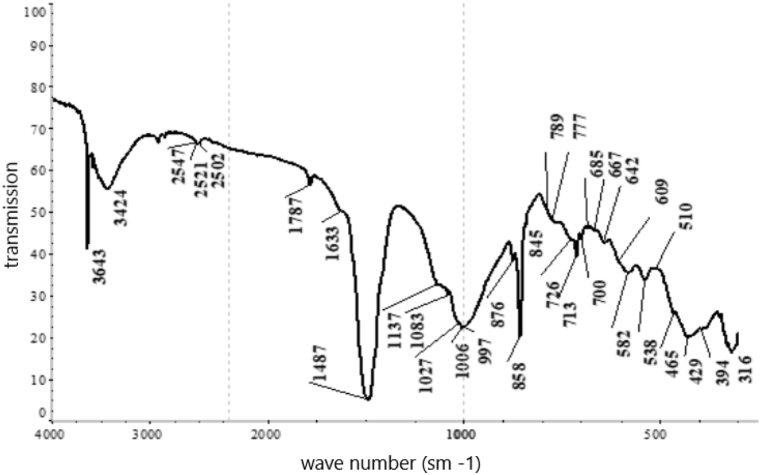
Infrared spectrum of the cake after leaching for 30 min.

**Fig. 16:**
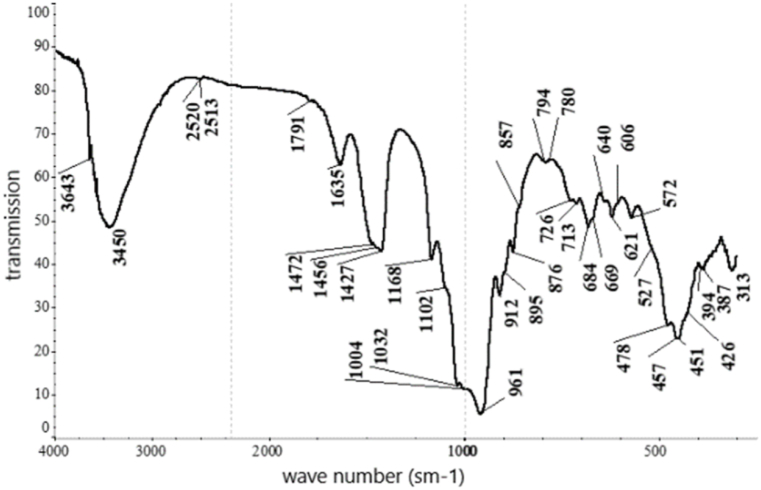
Infrared spectrum of the cake after leaching for 60 min.

**Fig. 17:**
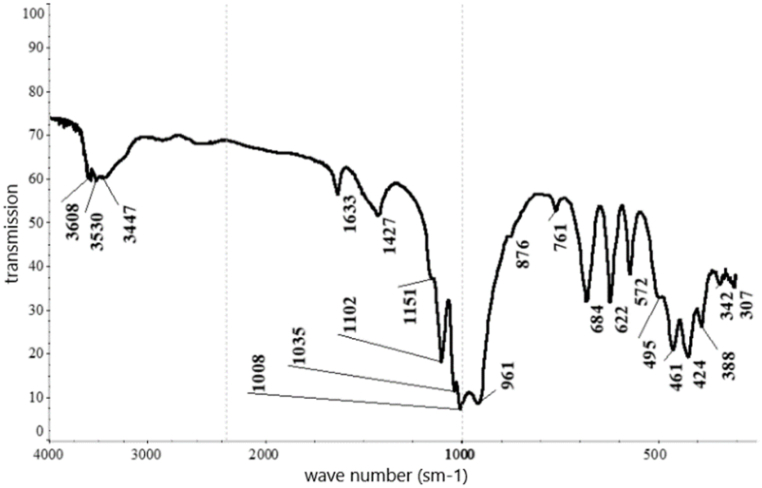
Infrared spectrum of the cake after leaching for 90 min.

**Fig. 18:**
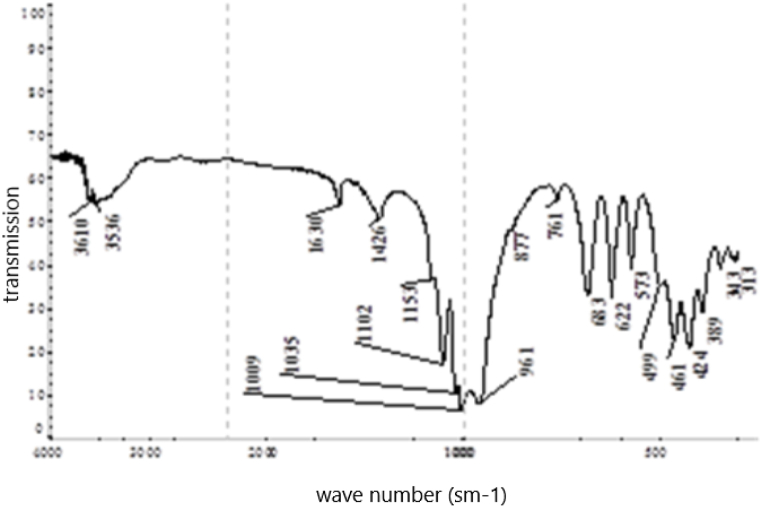
Infrared spectrum of the cake after leaching for 120 min.

Based on the results obtained from the XRF analysis, a leaching duration of 30 min was insufficient because 26.1 % of the cake consisted of unreacted portlandite. As the duration increased, the content of this phase diminished, and it completely disappeared after 90 min. Thus, this time interval for leaching was considered optimal based on the observed changes in the content of the specified phase. The phase composition of the cake samples was investigated by IRS analysis (see Table 6, Fig. 15, Fig. 16, Fig. 17, Fig. 18).

**Table 6:** Substantial composition of the cake samples by IRS analysis depending on duration.

Minerals detected	Leach cakes at leaching time, minutes			
	30	60	90	120
Ca(OH)_2_	+	+		
Microcline K[AlSi_3_O_8_]	+			
Aragonite CaCO_3_	+	+		
Albitite Na[AlSi_3_O_8_]	+			
Nepheline (Na; K)AlSiO_4_	+			
Calcite CaCO_3_	+	+		
Gypsum CaSO_4_·2H_2_O	+			
Valence vibrations νCa-O	+	+	+	+
Tobermorite Ca_4·5_Si_6_O_16_(OH)(H_2_O)_5_		+		
Tobermorite (unheated mineral, 14 Å)		+		
Cancrinite - (Na_6_(Al_6_Si_6_O_24_))(NaOH)_2_ (H_2_O)_6_		+	+	+
Beidellite - (Na,Ca)(Al, Mg)_2_ [AlSi_3_O_8_] × (OH)_2_∙n H_2_O		+		
Corundum α-Al_2_O_3_		+		
Corundum Al_2_O_3_		+		
Aluminium oxide γ-Al_2_O_3_		+		
Ankerite CaFe [CO3]2		+		
Group [CO_3_]^2-^			+	+

Peak positions of identified minerals are as follows.

Ca(OH)_2_: 3643 cm‾^1^.
Microcline K[AlSi_3_O_8_]: 1137, 1027, 1006, 777, 726, 642, 582, 538, 465 and 429 cm‾^1^
Aragonite CaCO_3_: 2547, 2521, 2502, 1787, 1487, 1083, 858, 713 and 700 cm‾^1^
Albite Na[AlSi_3_O_8_]: 789, 642, 609, 465 and 429 cm‾^1^
Nepheline (Na; K)AlSiO_4_: 997, 700, 582, 510 and 465 cm‾^1^
Calcite CaCO_3_: 876, 845 and 713 cm‾^1^
Gypsum CaSO_4_·2H_2_O: 1633, 1137, 1006 and 667 cm‾^1^

Peak positions of identified minerals are as follows.

Tobermorite Ca_5_Si_6_ (O; OH)_18_·5H_2_O: 1456, 1427, 1032, 961, 876, 713, 606 and 478 cm‾^1^
Tobermorite (unheated mineral, 14 Å): 1427, 961, 857 and 669 cm‾^1^
Cancrinite group mineral: 1102, 1032, 1004, 857, 684,621, 572, 457 and 426 cm‾^1^
Portlandite Ca(OH)_2_: 3643 and 451 cm‾^1^
Aragonite CaCO_3_: 2520, 1472, 857 and 713 cm‾^1^
Calcite CaCO_3_: 2513, 1791, 1427, 876 and 713 cm‾^1^
Beidellite - (Na,Ca)(Al, Mg)_2_ [AlSi_3_O_8_] × (OH)_2_∙n H_2_O: 1635, 1032, 912, 794, 780, 640, 527 and 478 cm‾^1^
Corundum α-Al_2_O_3_: 640, 606, 457 and 394 cm‾^1^
Corundum Al_2_O_3_: 640, 606 and 457 cm‾^1^
Aluminium oxide γ-Al_2_O_3_: 895 and 606 cm‾^1^
Ankerit CaFe [CO_3_]_2_: 1456, 876 and 726 cm‾^1^
Valence vibrations νCa–O: 313 cm‾^1^
The following may also be present:
Gypsum CaSO_4_·2H_2_O: 1004 and 669 cm‾^1^
CaSO_4_∙0,5H_2_O: 1102, 669 and 606 cm‾^1^
Coquimbite Fe_3_(SO_4_)_3_·9H_2_O: 1168, 1102, 669, 478, and 457 cm‾^1^

Peak positions of the identified minerals are as follows.

Cancrinite (Na_6_(Al_6_Si_6_O_24_))(NaOH)_2_ (H_2_O)_6_: 3608, 3530, 1633, 1102, 1035, 1008, 961, 761, 684,622, 572, 495, 461, and 424 cm‾^1^
[CO_3_]^2–^ group: 1427 and 876 cm‾^1^
Valence vibrations νCa–O: 342 and 307 cm‾^1^

The following may also be present.

Tobermorite (unheated mineral, 14 Å): 1427 and 961 cm‾^1^
Gypsum CaSO_4_·2H_2_O: 3608, 1151 and 1008 cm‾^1^

Cancrinite (Na_2_,Ca)_4_[CO_3_|(H_2_O)_0-8_| (AlSiO_4_)_6_]: 3610, 3536, 1630, 1035, 1009, 961, 761, 683,622, 573, 499, 461, and 424 cm‾^1^
[CO_3_]^2–^ group: 1426, 877 cm‾^1^
Valence vibrations νCa–O: 343 and 313 cm‾^1^
The following may also be present:
Tobermorite (unheated mineral, 14 Å):1426 and 961 cm‾^1^
Gypsum CaSO_4_·2H_2_O: 3610, 1153 and 1009 cm‾^1^

The IRS analysis data confirmed the findings from X-ray phase analysis since no potassium-containing phases were detected after leaching was conducted for more than 90 min. These results indicated that potassium transitioned into the solution phase during this extended leaching period.

Thus, to achieve the goal of selective potassium extraction in alkaline solution, the optimal leaching regime is as follows: the Na_2_O content of 240/dm^3^, a temperature of 280 °C and a duration of 90 min.

Chemical composition of the leaching cake under optimal conditions is as follows in wt.%: Al_2_O_3_ – 12.35; SiO_2_ – 27.83; Fe_2_O_3_ – 1.84; CaO – 15.765; Na_2_O – 14,44; and K_2_O – 0.25.

**Fig. 19:**
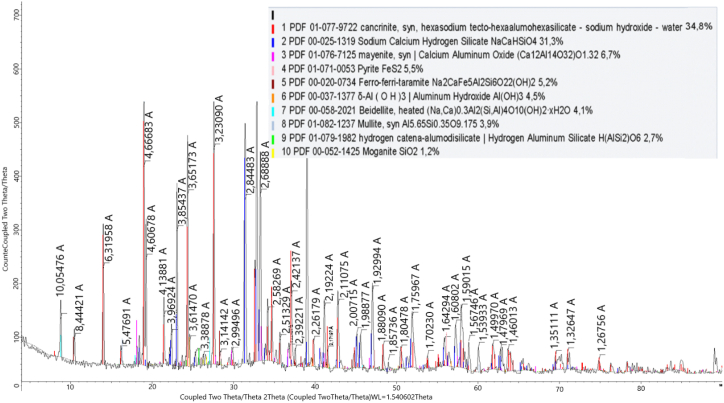
XRF phase spectra of the cake at a leaching time of 90 min.

The X-ray fluorescence (XRF) phase of the leach cake under optimum conditions is shown in Fig. 19.

Based on the results from the phase analysis of the initial and obtained products, the process of leaching of the nepheline syenite ore with the addition of calcium additive under optimal conditions can be shown in the form of the basic reactions associated with the decomposition of microcline and nepheline, with the formation of cancrinite and sodium calcium hydrosilicate, as follows:

(4) 3(K_2_O·Al_2_O_3_·6SiO_2_) + 20NaOH + CaSO_4_ + 9Ca(OH)_2_ → 3Na_2_O·3Al_2_O_3_·6SiO_2_·2NaOH·6H_2_O +6(Na_2_O·2CaO·2SiO_2_·H_2_O) + 3K_2_SO_4_ + 6H_2_O

3Na_2_O·3Al_2_O_3_·6SiO_2_·2NaOH·6H_2_O + Na_2_O·Al_2_O_3_ → Na_2_O·Al_2_O_3_·6SiO_2_ +2Ca(OH)_2_ +2NaOH (4)

(6) 2(Na_2_O·Al_2_O_3_·SiO_2_) +2NaOH + Ca(OH)2 → Na_2_O·2CaO·2SiO_2_·H_2_O + 2Na_2_O·Al_2_O_3_·2H_2_O

Thus, by leaching the nepheline syenites, the goal of the selective extraction of potassium into solution was achieved. At the same time, the minimum extraction of aluminium and silicon was achieved. The minimum extraction of aluminium and silicon simultaneously was achieved. Leach cakes did not contain potassium-containing compounds. CaSO_4_ was added to the calcium additive to obtain potassium salts in the form of potassium sulfate. By separating potassium beforehand, we could eliminate its negative impact on the alumina extraction. This selective extraction of potassium is a distinctive feature of our complex technology developed for nepheline syenite processing.

## 4 Conclusions

The purpose of this study was to selectively separate potassium alkali from sodium alkali, aluminium, and silicon during the initial stage with a fractional addition of calcium additive during the hydrothermal leaching process for the complex processing of nepheline syenites. The optimal technological conditions for the leaching operation were determined. These optimal conditions consisted of the following: a sodium oxide solution concentration of 240 g/dm³, a leaching duration of 90 min, a temperature of 280 °C, and the addition of a calcium additive at 70 % of the molar ratio of CaO:SiO_2_ = 1; under these conditions, a potassium oxide recovery in the solution ranging from 92.13 % to 93.91 % was achieved. The potassium-containing alkaline leaching solution served as a product for obtaining potassium sulfate through the crystallization method, while the leach cake was utilized as a product for the hydrothermal production of alumina and calcium silicate.

## Data availability statement

All data required to support this study is already mentioned in manuscript.

## CRediT authorship contribution statement

**Nazym Akhmadiyeva:** Writing – original draft, Conceptualization. **Sergey Gladyshev:** Writing – review & editing, Writing – original draft. **Rinat Abdulvaliyev:** Writing – review & editing, Conceptualization. **Bulat Sukurov:** Writing – review & editing, Formal analysis. **Leila Amanzholova:** Formal analysis.

## Declaration of competing interest

The authors declare that they have no known competing financial interests or personal relationships that could have appeared to influence the work reported in this paper.

## References

[1] Abramov V., Alekseev A., Badalyats H. (1990). Complex processing of nepheline-apatite raw materials. Moscow. Metallurgy.

[2] Jena S.K. (2020). A review on potash recovery from different rock and mineral sources. Mining, metallurgy and exploration.

[3] Smeshnikova E.V., Andreeva E.D., Kononova V.A., Yashina R.M. (1978). Nepheline Raw Materials.

[4] Jena S.K., Dhawan N., Rao D.S., Misra P.K., Mishra B.K., Das B. (2014). Studies on extraction of potassium values from nepheline syenite. Int. J. Miner. Process..

[5] Majumder A.K., Govindarajan B., Sharma T., Ray H.S., Rao T.S. (1995). An empirical model for chloridizing-roasting of potassium in glauconitic sandstone. Int. J. Miner. Process..

[6] Yuan B., Li C., Liang B., Li L., Yue H., heng S.H., Ye L., Xie H. (2015). Extraction of potassium from K-fieldspar via CaCl_2_ calcination route. Chin. J. Chem. Eng..

[7] Bagani M., Efthynios B., Dimitrios P. (2021). Nepheline syenite as an alternative source for aluminum production, nepheline syenite as an alternative source for aluminum production. Minerals.

[8] (2001). V.N. Lebedev Patent RU 2215690 Method of Processing Nepheline Concentrate 2001112507/12 of 07.05.

[9] Kangal M., Bulut G., Yesilyurt Z., Basturkcu H., Burat F. (2019). Characterization and production of Turkish nepheline syenites for industrial applications. Physicochem. Probl. Miner. Process..

[10] Ma H., Yang J., Su S., Liu M., Zheng H., Wang Y., Qi H., Zhang P., Yao W. (2015). 20 years advances in preparation of potassium salts from potassic rocks: a review. Acta Geol. Sin..

[11] Naimov N.A., Aminjoni G., Ruziev J.R., Rafiev R.S., Boboev H.E., Safiev H. (2018). Physicochemical aspects of processing staurolite-muscovite shale by sulfatization method. Reports of the Academy of Sciences of the Republic of Tajikistan.

[12] Shepelev I.I., Algebraistova N.K., Sakhachev A.Y., Zhizhaev A.M., Prokopyev I.V. (2017).

[13] Bo Y., Li C., Liang B., Lu L., Yue H., Sheng H., Ye L., Xie H. (2015). Extraction of potassium from K-feldspar via CaCl_2_ calcination route. Energy, Resources and Environmental Technology.

[14] Ogol V.I., Yagin V.P. (2012).

[15] Zhang H., Sun D., Bao H. (2012). The extraction of potassium from feldspar by molten salt leaching method with composite additives. Adv. Mater. Res..

[16] Coulson I.M., Chambers A.D. (1996). Patterns of zonation in rare-earth-bearing minerals in nepheline syenites of the North Qôroq center, South Greenland. Can. Mineral..

[17] Ciceri D., Oliveira M., Stokes R., Skorina T., Allanore A. (2017). Characterization of potassium agrominerals: correlations between petrographic features, comminution and leaching of ultrapotassic syenites. Miner. Eng..

[18] Gorbunova E.S., Zakharov V.I., Fedorov S.G., Alishkin A.R., Matveev V.A., Mayorov D.V. (2008).

[19] Kazakov V.G., Lipin V.A., Matveev V.A., Mayorov D.V., Korovin V.N. (2013). Mutual influence of parameters of hydrochemical processing of nepheline on alumina. J. Appl. Chem..

[20] Kovzalenko V.A., Myltykbaeva L.A., Tastanov E.A., Beisembekova K.O. (2006). Processing of aluminosilicate raw materials by hydrochemical method with preliminary chemical enrichment. Journal of energy technology and resource saving.

[21] E.A. Tastanov, R.A. Abdulvaliev, U.Zh Sadyralieva, S.V. Gladyshev, K.O. Beisembekova, V.A. Pozmogov, Patent KZ 29829 Method of Chemical Enrichment of Nepheline (In Russian)..

[22] Unkear A.T., Budakoglu M., Doner Z. (2023). The evolution of the REE-bearing özvatan nepheline syenite-carbonatite complex, Central Turkey: mineralogical, geochemical, and stable isotopic approaches. Minerals.

[23] Abdulvaliyev R., Akhmadiyeva N., Gladyshev S., Samenova N., Kolesnikova O., Mankesheva Olimpiada (2023). Behavior of calcium compounds under hydrothermal conditions during alkaline leaching of aluminosilicates with the synthesis of fillers for composites. Journal of Composites Science.

[24] Sazhin S. (1988).

